# Is the current decline in malaria burden in sub-Saharan Africa due to a decrease in vector population?

**DOI:** 10.1186/1475-2875-10-188

**Published:** 2011-07-13

**Authors:** Dan W Meyrowitsch, Erling M Pedersen, Michael Alifrangis, Thomas H Scheike, Mwelecele N Malecela, Stephen M Magesa, Yahya A Derua, Rwehumbiza T Rwegoshora, Edwin Michael, Paul E Simonsen

**Affiliations:** 1 Section of Health Services Research, Department of Public Health, University of Copenhagen, Øster Farimagsgade 5B, DK-1014, Copenhagen K, Denmark; 2 DBL-Centre for Health Research and Development, Faculty of Life Sciences, University of Copenhagen, Thorvaldsensvej 57, DK-1871, Frederiksberg C, Denmark; 3 Centre for Medical Parasitology, Department of International Health, Immunology and Microbiology, University of Copenhagen, Øster Farimagsgade 5B, DK-1014, Copenhagen K, Denmark; 4 Section of Biostatistics, Department of Public Health, University of Copenhagen, Øster Farimagsgade 5B, DK-1014, Copenhagen K, Denmark; 5 National Institute for Medical Research, P.O. Box 9653, Dar-es-Salaam, Tanzania; 6 RTI International, P.O. Box 6201, Kigali, Rwanda; 7 National Institute for Medical Research, Amani Medical Research Centre, P.O. Box 81, Muheza, Tanzania; 8 Department of Biological Sciences, Eck Institute for Global Health, 349 Galvin Life Sciences Center, University of Notre Dame, Notre Dame, IN 46556-0369, USA

## Abstract

**Background:**

In sub-Saharan Africa (SSA), malaria caused by *Plasmodium falciparum *has historically been a major contributor to morbidity and mortality. Recent reports indicate a pronounced decline in infection and disease rates which are commonly ascribed to large-scale bed net programmes and improved case management. However, the decline has also occurred in areas with limited or no intervention. The present study assessed temporal changes in Anopheline populations in two highly malaria-endemic communities of NE Tanzania during the period 1998-2009.

**Methods:**

Between 1998 and 2001 (1st period) and between 2003 and 2009 (2nd period), mosquitoes were collected weekly in 50 households using CDC light traps. Data on rainfall were obtained from the nearby climate station and were used to analyze the association between monthly rainfall and malaria mosquito populations.

**Results:**

The average number of *Anopheles gambiae *and *Anopheles funestus *per trap decreased by 76.8% and 55.3%, respectively over the 1st period, and by 99.7% and 99.8% over the 2nd period. During the last year of sampling (2009), the use of 2368 traps produced a total of only 14 Anopheline mosquitoes. With the exception of the decline in *An. gambiae *during the 1st period, the results did not reveal any statistical association between mean trend in monthly rainfall and declining malaria vector populations.

**Conclusion:**

A longitudinal decline in the density of malaria mosquito vectors was seen during both study periods despite the absence of organized vector control. Part of the decline could be associated with changes in the pattern of monthly rainfall, but other factors may also contribute to the dramatic downward trend. A similar decline in malaria vector densities could contribute to the decrease in levels of malaria infection reported from many parts of SSA.

## Background

Approximately half of the world's population is at risk of malaria, and an estimated 243 million infected cases resulted in nearly 863,000 deaths in 2008 [1]. In sub-Saharan Africa (SSA), where 91% of all malaria-related deaths take place, malaria is estimated to result in an annual loss of 35.4 million Disability Adjusted Life Years with 85% of the deaths amongst children below five years of age. In SSA, around 40% of all public health spending is related to malaria [2].

Despite these distressing records, reductions in the numbers of malaria cases and malaria-related deaths by up to 50% over the past decade have been reported from several high burden African countries [3], including Eritrea, Rwanda, Zanzibar [1], Pemba [4], Tanzania mainland [5], Kenya [6] and Zambia [7]. The declining infection rates and overall disease burdens as well as reduction in asymptomatic carriers are considered to be a consequence of improved quality of health systems, including improved case management, such as enhanced diagnostics and implementation of highly effective anti-malarial drugs. Large scale investments in intervention programmes specifically aimed at achieving high coverage of bed nets, campaigns of indoor residual spraying (IRS) and implementation of intermittent presumptive treatment (IPT) in vulnerable groups, have further reduced the malaria burden significantly [1].

The scale-up of malaria control interventions in high-endemic countries has without doubt contributed to the observed decline in malaria cases and deaths. However, other factors not related to intervention could potentially have an impact on mosquito vectors, and thereby reduce transmission, which subsequently will result in reductions in number of infected cases. Among these factors are urbanization, changes in agricultural practices and land use, and economic development resulting in e.g. improved housing construction [6]. Global climatic changes resulting in changes in rainfall, humidity and temperature may also impact malaria vectors [8-10]. Despite the relevance of these multiple determinants and their potential roles in the declining malaria burdens, the majority of field studies reporting changing malaria epidemiology focus exclusively on the change in the occurrence of human infections and disease. In this respect, there have also been only limited research efforts invested in systematic data collection and mapping of long-term trends in vector densities and other vector related aspects of transmission in the areas where declining malaria infection and disease burdens have been reported.

Tanga Region in north-eastern Tanzania is among the areas in SSA where a significant reduction in the prevalence and burden of malaria recently has been observed [5]. In the following, the temporal change in mosquito vector densities in the absence of organized vector control activities in two rural communities in this area during the period 1998-2009 is reported and discussed. The results in relation to an assessment of the effect of a drug based intervention targeting lymphatic filariasis in these communities during a part of this period have previously been published [11,12], but here the attention focuses on temporal patterns observed in catches of the primary mosquito vectors, viz. *Anopheles gambiae *and *Anopheles funestus*, responsible for malaria transmission in this region.

## Methods

### Study area

The study was performed in two rural communities, Masaika (5° 16' 0'' S, 38° 49' 60'' E) and Kirare (5° 15' 4'' S, 39° 1' 47'' E), located 25 and two kilometres, respectively, from the Indian Ocean in Tanga Region, Tanzania. *Plasmodium falciparum *malaria is highly endemic in the area and out of an annual under-five year mortality rate of 158/1,000 births (1992-1993), malaria has previously been estimated to account for one out of three child deaths [13]. The area is considered to normally have two annual rainy seasons; the 'long rains' in March-June and the 'short rains' in October-November. The majority of the inhabitants live in mud-walled houses thatched with dried coconut leaves. The main occupation in both communities is subsistence farming, whereas small-scale fishing contributes to the household income in one section of Kirare. At the end of each of the two study periods, the communities had populations of approximately 1,000 and 1,300 inhabitants, respectively. For further details regarding the characteristics of the two communities, please see [12,14].

Initially, both of these study communities were selected as study sites for research projects focusing on the epidemiology and control of lymphatic filariasis (*Wuchereria bancrofti *infection). The intervention targeting lymphatic filariasis was based on mass drug administration using anti-filarial drugs and did not include any type of vector control measure. Hence, during the study period, there have been no organized attempts to control the mosquito vector populations, neither with indoor or outdoor insecticide spraying, nor via environmental management targeting mosquito breeding sites. During the period 6^th ^July 1998 to 30^th ^November 2001, mosquitoes were collected in Masaika. Following an intermediate period from 1^st ^December 2001 to 9^th ^November 2003 without data collection, collection of mosquitoes commenced again in Kirare from 10^th ^November 2003 to 31^st ^December 2009. For more details regarding the study designs, please see [11,12,14-16].

At the time of the study in Masaika, and during the early part of the study in Kirare, very few households used bed nets. In April 2010 (four months after cease of mosquito collection for the present study), a survey in Kirare indicated that the number of bed nets (all types) relative to the number of inhabitants was 27.6%, and 66.4% of all households had a bed net (all types). Sixty-one out of 351 households (17.4%) were found to have at least one insecticide-treated net (ITN). The number of ITNs relative to the number of inhabitants, however, was just 6.6%. Use of mosquito coils and other mosquito prevention measures are very rare.

The primary vectors of malaria in sub-Saharan Africa are freshwater breeding members of the *Anopheles gambiae *s.l. complex and *Anopheles funestus *[17]. The predominant indoor man-biting mosquitoes in the Tanga Region are *An. gambiae *s.l., *An. funestus *and *Culex quinquefasciatus *[11,12,15]. *Anopheles gambiae *and *An. funestus *breed in clear water, the former typically in stagnant temporary pools without vegetation and the latter in more permanent water bodies often with some vegetation where the larvae can tolerate some water movements. *Culex quinquefasciatus *can tolerate water with a very high content of organic matter and are typically found breeding in cesspools, pit-latrines and septic tanks [18].

### Mosquito collection and identification

Among the approximate 285 and 350 households in Masaika and Kirare, respectively, 50 households were randomly selected in each community using a cluster sampling technique as described previously [15]. If the selected household only had one inhabitant, the nearest household with at least two inhabitants was selected instead. Mosquitoes were sampled from each of the 50 selected households in each community once weekly for the mentioned period, using one CDC light trap/household [17]. In this area, the CDC light trap effectively collects host-seeking indoor night-biting *Anopheles *and *Culex *mosquitoes, and the number of mosquitoes caught in one light trap is approximately equal to two-thirds of the number caught by one person acting as bait in human landing collection [19]. In each community, mosquito collection took place in 10 households on each weekday night. Each individual sleeping in a room with a light trap was provided with a 2-mm-mesh polyester un-impregnated bed net measuring 1 × 2.5 × 2 m. In each study household, the light trap was placed beside an occupied bed net. In both communities, a trained member of the household supervised by a local field assistant turned the light trap on at 18.00 hours and turned off at 06.00 hours the following morning after the collection bag had been closed with a string. The local field assistant transferred the mosquitoes to a labeled paper cup covered with netting material, which held a cotton pad soaked in 10% glucose. The cups were placed in a cool box and transported to the laboratory in Tanga Town, where the mosquitoes were anaesthetized with ethyl acetate, sorted, identified by morphological characteristics and counted. In the present paper, the categorization of mosquito species groups was limited to *An. gambiae s.l*., *An. funestus *and *Cx. quinquefasciatus*. For further details regarding the sampling and identification of mosquitoes, please see [11,12,15].

### Rain fall data

The daily rain fall data from the Maji Depot Tanga Rainfall station (Ministry of Water and Irrigation, Tanzania) in the period June 1^st ^1998 to December 31^st ^2010 were kindly provided by the Director of the rainfall station, Mr. Fundi. The rainfall station is located in the centre of Tanga Town (5 4' 58" S, 39 5' 21" E) approximately 35 and 20 kilometres from Masaika and Kirare, respectively.

### Data analysis

The daily average number of each of the three mosquito species per trap was calculated by dividing the total number of each species caught with the number of traps used on the specific day. These data were entered in Access and later transferred to SPSS (PASW 18, version 18.0.0) for generation of simple frequency tables. Analyses of mosquito and rainfall time series from each study location were based on monthly aggregates derived for these variables. Longer-term trends in each rainfall/mosquito series were assessed using the non-parametric seasonal Mann-Kendall test that takes account of any seasonality in these data [20]. Cumulative periodogram analysis was also applied to each rain fall series in order to detect differences in important frequencies/periodicities between the two study locations or time periods [21]. Finally, the temporal association between monthly mosquito counts and mean monthly rainfall and month itself was investigated using Poisson generalized additive mixed models (GAMMs) with a AR-1 autocorrelation function to take account of serial correlation in the data [22]. The total number of traps used per month was employed as an offset in the fitted models.

## Results

Mosquitoes were systematically collected for a total period of nine years in the two rural communities. Overall, mosquitoes were collected for a total of 2,407 weekdays excluding 85 intermediate weekdays (representing less than 4% of the originally planned days of collection), when mosquitoes were not collected due to holidays, funerals and logistic problems. Due to occasional absence of habitants in the selected catching households, light traps were some times not installed in all 10 households on each weekday. During the weekdays where collection of mosquitoes took place, the mean number of traps was 8.8. On 1252 collection days (52.0% of all collection days), mosquito collection took place in all 10 households. Data on the content of 21156 light traps are included in analysis of the present study.

**Figure 1:**
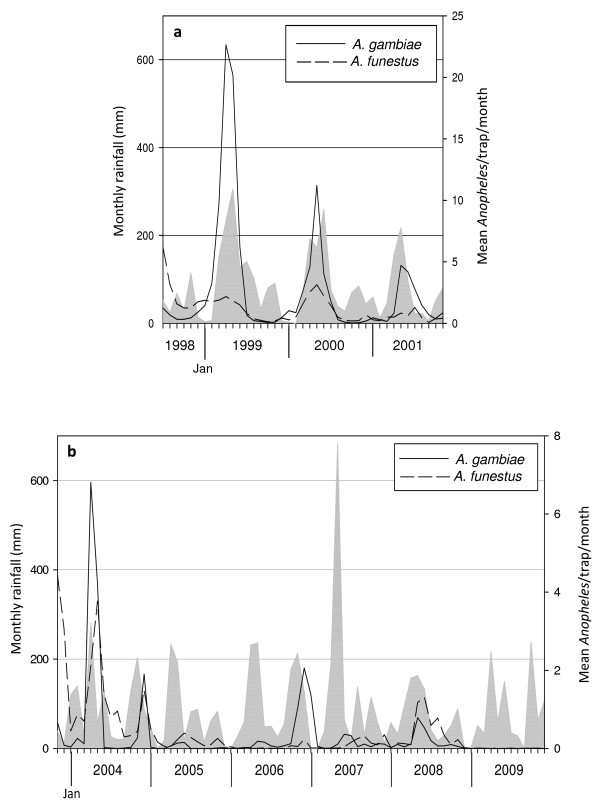
**The prevalence of in-door caught *Anopheles gambiae *and *An. funestus *and monthly rainfall pattern in Masaika village, 1998-2001 (a) and Kirare, 2004-2009 (b), Tanga region, Tanzania**. The prevalence is shown as mean number of *An. gambiae *or *An. funestus *caught per CDC light trap per month. Note that this parameter has different scale on the y-axis in the two sub-figures. The monthly rainfall has been recorded at the Maji Depot Tanga Rainfall station (Ministry of Water and Irrigation, Tanzania) and is shown as a gray area plot.

**Figure 2:**
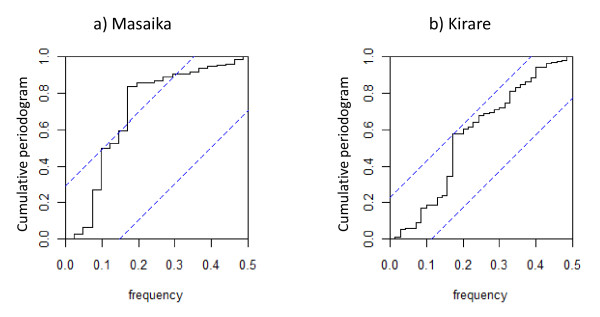
**Cumulative periodogram for Masaika (a) and Kirare (b) monthly rainfall series data (solid lines)**. Dashed lines indicate the upper and lower limits for the null hypothesis that the respective series is purely noise.

An overview of rainfall, number of traps used and number of mosquitoes sampled in Masaika (first period, 1998-2001) and in Kirare (second period, 2004-2009) are presented in Table 1 and 2, respectively. In order to allow a comparison of annual collections of equal periods, precipitation and mosquito data collected during the periods 6^th ^July - 30^th ^November 1998 (Masaika) and 10^th ^November 2003 - 31^st ^December 2003 (Kirare) are not presented in the tables. These data are, however, included in the statistical analyses and subsequent presentation of results (Figures 1 and 2). As shown in Table 1 the pre-dominant Anopheline species at the start of the data collection period in Masaika was *An. gambiae*, whereas in Kirare (Table 2) both *An. gambiae *and *An. funestus *occurred with almost the same frequency at start of data collection. From the initial period to last period of sampling in Masaika, the average number of *An. gambiae, An. funestus *and *Cx. quinquefasciatus *collected per trap decreased by 76.8%, 55.3% and 64.0%, respectively (Table 1 and Figure 1a). The monthly change in mosquito numbers in Masaika (first period) is shown in Figure 1a; statistical analysis of these data using the fitted GAMM model indicates that the declines observed in the Figure were statistical significant for all three mosquito species (p < 0.001 for both Anopheles; p = 0.011 for Culex, respectively). In Kirare, the equivalent decreases for *An. gambiae, An. funestus *and *Cx. quinquefasciatus *were 99.7%, 99.8% and 38.8%, respectively (Table 2 and Figure 1b). However, while the longitudinal decline in *An. gambiae *assumed significance (p = 0.049), the declines in number of *An. funestus *and *Cx. quinquefasciatus *per trap were either only borderline or found not to be significant (p = 0.089 and p = 0.534, respectively). During the last year of mosquito collection in Kirare (2009), the use of 2368 traps produced a total of only nine *An. gambiae *and five *An. funestus *mosquitoes.

**Table 1:** Rainfall, periods of sampling, number of traps and number of mosquitoes collected in Masika during the period 1998-2001

Year	1998/1999	1999/2000	2000/2001
Period of mosquito sampling (day/month/year)	1/12/1998 - 30/11/1999	1/12/1999 - 30/11/2000	1/12/2000 - 30/11/2001
Total rainfall for period of mosquito sampling (mm)	1,292	996	815
No. weekdays of mosquito sampling	260	272	257
No. of traps used (mean no. of traps per weekday)	2,204 (8.48)	1,877 (7.16)	1,562 (5.98)
Total no. of collected *An. gambiae * (average no. per trap)	12,465 (5.66)	4,484 (2.39)	2,125 (1.36)
Total no. of collected *An. funestus * (average no. per trap)	2,716 (1.23)	2,145 (1.14)	858 (0.55)
Total no. of collected *Culex quinquefasciatus * (average no. per trap)	3,304 (1.50)	1,648 (0.88)	837 (0.54)

Data collected during the periods 6^th ^July - 30^th ^November 1998 are not presented in the table but are included in the statistical analysis.

**Table 2:** Rainfall, periods of sampling, number of traps and number of mosquitoes collected in Kirare during the period 2004-2009

Year	2004	2005	2006	2007	2008	2009
Period of mosquito sampling (day/month/year)	1/1/2004- 31/12/2004	1/1/2005- 31/12/2005	1/1/2006- 31/12/2006	1/1/2007- 31/12/2007	1/1/2008- 31/12/2008	1/1/2009- 31/12/2009
Total rainfall for period of mosquito sampling (mm)	1,243	843	1,254	1,348	870	987
No. weekdays of mosquito sampling	253	245	247	250	246	248
No. of traps used (mean no. of traps per weekday)	2,428 (9.27)	2,376 (9.14)	2,332 (9.00)	2,386 (9.14)	2,310 (8.85)	2,368 (9.07)
Total no. of collected *An. gambiae * (average no. per trap)	2,657 (1.0943)	88 (0.0370)	686 (0.2942)	533 (0.2234)	410 (0.1775)	9 (0.0038)
Total no. of collected *An. funestus * (average no. per trap)	2,725 (1.1223)	470 (0.1978)	76 (0.0326)	272 (0.1140)	939 (0.4065)	5 (0.0021)
Total no. of collected *Culex quinquefasciatus * (average no. per trap)	4,081 (1.6808)	5,507 (2.3177)	6,171 (2.6462)	5,403 (2.2645)	4,115 (1.7814)	2,396 (1.0118)

Data collected during the periods 10^th ^November 2003 - 31^st ^December 2004 are not presented in the table but are included in the statistical analysis.

Whereas the GAMM modeling showed that for the first study period (Masaika), the observed decline in *An. gambiae *numbers over the entire study duration was positively related to longitudinal changes in rainfall (p = 0.002), the numbers of *An. funestus *and *Cx. quinquefasciatus *were apparently not (p = 0.924 and p = 0.071, respectively). By contrast, for the second period (Kirare), while *Cx. quinquefasciatus *numbers were found to positively related with rainfall fluctuations (p = 0.023), the declines observed for both *An. gambiae and An. funestus *appeared unrelated to this climatic variable (p = 0.083 and p = 0.089, respectively).

With respect to rainfall changes *per se*, the results indicated that monthly rainfall during the first period of sampling (Masaika) exhibited a statistically significant decreasing trend (seasonal Mann-Kendall tau = -0.412; p = 0.012), whereas it appeared unchanged over the second period (Kirare) (tau = -0.047; p = 0.011). Figure 2 depicts the cumulative periodograms obtained for the monthly rainfall series in Masaika and Kirare respectively. The plot for the Masaika data (Figure 2a) shows the estimated periodogram crossing the upper limit of the null hypothesis that the series is purely noise at a frequency of 0.088 (i.e. at around 12 months) with another vertical step occurring at a frequency of 0.155 or at around 6 months. These frequencies correspond to the existence of annual and 6-monthly peaks in monthly rainfall or two rainy seasons in this location (Figure 2a). By direct contrast, the cumulative periodogram for Kirare (2^nd ^period) remained bounded within the upper and lower boundaries of the null hypothesis (Figure 2b), suggesting the absence of a well defined periodicity in the observed monthly rainfall pattern in this location (see Figure 1b).

## Discussion

The present study clearly revealed, in the absence of organized vector control activities, a dramatic temporal decline in the density of malaria mosquito vectors in a SSA rural area with a previous history of high *P. falciparum *malaria endemicity. The results indicated that the indoor density of the two main vectors of *P. falciparum *malaria, *An. gambiae *and *An. funestus*, declined considerably during both study periods, which covered a total time span of more than 9 years. The overall decline in the number of Anopheline mosquitoes was within the range of 55-77% over the first study period (1998-2001), whereas the trend continued and became more pronounced over the subsequent period (2003-2009). Hence, during the last year of mosquito collection in Kirare, the annual numbers of collected *An. gambiae *and *An. funestus*, as compared to 2004, had declined by more than 99%. Despite the annual employment of more than 2300 traps, a total of only 14 Anopheline mosquitoes were collected in 2009. A valid comparison of the two study periods should be performed with caution, since the data were collected in different communities, which, despite their relative proximity to each other may differ in terms of local factors of relevance for mosquito vector capacity and population dynamics, including variations in quality and number of breeding sites, microclimatic conditions, housing structures or environmental factors.

The *P. falciparum *infection rates in humans were not assessed in the study communities during the two study periods. However, an almost complete absence of indoor exposure to Anopheline mosquitoes, as observed in Kirare in 2009, would most likely result in a rapid and pronounced decline in intensity of transmission, and thereby the prevalence of malaria infection, morbidity and mortality among the study inhabitants.

Recent reports and publications have described pronounced reductions in the prevalence of *P. falciparum *infections and malaria-related disease burdens among inhabitants in several African countries over the past decade [1,4-7]. Interestingly, the study from Pemba, Tanzania, an island in close proximity to Tanga Region, suggested that malaria transmission had started to decrease *prior to *the onset of a control programme implemented during the period 2003-2005 [4]. Of specific relevance to the present study are also longitudinal investigations carried out in two rural communities (lowland and highland) in Korogwe District, Tanga Region, Tanzania - an area located less than 70 kilometre from Masaika and Kirare [5]. During the period 2003-2007, the *P. falciparum *prevalence decreased dramatically from 78.4% to 24.0% in the lowland community, and from 24.7% to 6.5% in the highland community, respectively. Likewise, the incidence of febrile malaria episodes in the two communities decreased by almost 85% during the same period. Temporal changes in mosquito vector densities were not assessed by the authors and specific reasons for the observed decline in *P. falciparum *infections and related morbidity could not be identified. When considering the proximity to the communities included in the present study, the results suggest that it is likely that the decline in malaria infection and disease burden in those communities could have been a result of a decline in the Anopheline population of perhaps a similar magnitude as observed in the present study. If this assumption is correct, the findings suggest that at least in some areas in Africa, the decline in prevalence of human *P. falciparum *infections and malaria-related disease is driven by a natural decline in the occurrence of mosquito vectors. In tropical Africa, where mosquito abundance often follows the rainy season, precipitation can be regarded as the primary climatic determinant for variation in Anopheline population size. However, the results suggest that this linkage to rainfall may be related in a complex fashion to the patterns of rainfall experienced in a location as well as the population dynamics and breeding habits of the vector species in question. Thus, while declines in *An. gambiae *numbers in Masaika were related to a declining trend in mean monthly rainfall, variations in none of the Kirare mosquito populations appeared to be related to changes in mean monthly rainfall. The present analysis has highlighted that one intriguing possibility that may underlie the decrease in *An. gambiae *populations in particular may arise as an outcome of the change from a regular seasonal rainfall pattern as observed in period 1 (Masaika) to a more noisy or variable temporal pattern observed in Kirare. This suggests that climate change leading to the disruption of stable seasonal rainfall in a location could result in highly dynamic vector population dynamics that might increase the probability of mosquito extinction [23].

This conclusion appears to be further supported by the fact that there has been no organized attempt to control mosquitoes using insecticides or environmental management in the two study communities. During the first study period only very few inhabitants in Masaika used a bed net, while in Kirare, although bed nets were rarely used in the beginning, a survey carried out after the end of the study indicated that the prevalence of bed nets reached 27% and some inhabitants also used ITNs (6.6%). The use of ITNs may have resulted in some decline in the indoor density of Anopheline mosquitoes and could have triggered a change in biting/resting behavior including a shift from an indoor to an outdoor environment [24]. The sibling species composition within the *An. gambiae *complex and the *An. funestus *group was not investigated in the present study. It has been suggested that a specific bed net coverage will result in a higher reduction in transmission by *An. gambiae *ss as compared to reduction in transmission by the more zoophagic and exophilic *An. arabiensis *[25]. However, even if all the *An. gambiae *in the present study were *senso stricto*, it is not likely that ITNs used by less than 7% of inhabitants (as seen during the last year of the study) would result in a 99% decrease in the indoor Anopheline mosquito populations [25].

The decline in mosquito numbers may also be a consequence of changes in socio-ecological conditions in the study area (e.g. changes in temperature, ability for water to pool, deforestation or land-use [8], change in the use of agricultural pesticides or insecticide-like compounds not directly applied for targeting malaria vectors [26], improved house constructions [27] or changes related to agricultural activities [28,29]). An increase in predatorily pressure on the mosquito population [30] (e.g. birds or invertebrates) or an insect pathogen that specifically targeted mosquitoes, e.g. a bacterial [31], viral [32] or fungi infection [33], could also potentially have induced the observed declines. Regardless of the actual cause, the marked decline in the density of Anophelines followed by their almost complete absence at the end of the second study period suggests that the causing factor or factors have placed an extreme pressure on these mosquitoes.

It is interesting to notice that in neither of the two study communities was the population of *Cx*. q*uinquefasciatus *affected with the same magnitude as compared to the two species of Anopheline mosquitoes. This difference suggests that either the *Culex *mosquitoes were less sensitive to the causing factor due to direct species-specific differences or that their pre-dominant breeding sites (organic polluted water bodies, pit latrines) were less affected by the changes as compared to the breeding sites of the Anopheline mosquitoes.

Assuming that the intensity of *P. falciparum *transmission has decreased to very low levels within the last five years, it is possible that an increasing proportion of inhabitants in this study area have only been marginally exposed to infections during this period. This would affect their natural acquisition of immunity, which is of specific importance for children who have been born and raised in the community within the last five years [34]. In a prospective scenario, where the factors which have suppressed the Anopheline mosquito population will cease to play a role, immunologically naïve or partly naïve children and adults may thus have a significant risk of severe malaria related morbidity and death [34]. In order to monitor and predict the risk of sudden epidemics it is, therefore, of outmost importance to identify the underlying causative factor or factors. The findings suggest that the pronounced decline in malaria mosquito vectors over the study periods is not a consequence of bed net use or indoor residual spraying. Although there may be an intriguing link to recent changes in rainfall patterns that requires further investigation, other potential explanations for the observed decline in vector populations in the two study communities should be explored, including an assessment of the potential role of changes in temperature, the ability for water to pool, agricultural activities, land use, vegetation, sibling species composition and impact of toxic substances and insect pathogens. It is likely that similar pronounced declines in malaria vector densities have contributed and still contribute to the decrease in levels of malaria infections and malaria disease seen in other geographical areas of SSA. Health professionals and researchers are strongly encouraged to initiate a systematic collection of the relevant mosquito vectors in order to monitor and assess the causative role of declining malaria vector populations in areas where decreasing levels of malaria infection and disease burden have been reported.

## Competing interests

The authors declare that they have no competing interests.

## Authors' contributions

DWM, MA, EMP and PES developed the idea for this paper. The text was drafted by DWM, with contributions from MA, PES, EMP and EM. Statistical data analyses were performed by THS and EM. The original studies, which provided the entomological data for this paper, were designed and carried out by PES, EMP, DWM, SMM, RTR, YAD, MNM and EM. All authors (except RTR, who passed away in 2006) read and approved the final manuscript.
